# Relationship between organizational structure and creativity in teaching hospitals

**Published:** 2014-07

**Authors:** RITA REZAEE, SAADAT MARHAMATI, PARISA NABEIEI, RAHELEH MARHAMATI

**Affiliations:** 1 Quality improvement in Clinical Education Research Center, Shiraz University of Medical Sciences, Shiraz, Iran;; 2 Shiraz University of Medical Sciences, Shiraz, Iran

**Keywords:** Organization, Management, Hospital

## Abstract

**Introduction: **Organization structure and manpower constitute two basic components of anorganization and both are necessary for stablishing an organization. The aim of this survey was to investigate the type of the organization structure (mechanic and organic) from viewpoint of senior and junior managers in Shiraz teaching hospitals and creativity in each of these two structures.

**Methods: **In this cross-sectional and descriptive-analytic study, organization structure and organizational creation questionnaires were filled out by hospital managers. According to the statistical consultation and due to limited target population, the entire study population was considered as sample. Thus, the sample size in this study was 84 (12 hospitals and every hospital, n = 7). For data analysis, SPSS 14 was used and Spearman correlation coefficient and t-test were used.

**Results: **Results showed that there is a negative association between centralization and complexity with organizational creation and its dimensions. Also there was a negative association between formalization and 4 organizational creation dimensions: reception change, accepting ambiguity, abet new view and less control outside (p=0.001).

**Conclusion: **The results of this study showed that the creation in hospitals with organic structure is more than that in hospitals with mechanic structure.

## Introduction

All organizations require new ideas as well as exquisite and fresh opinions in order to survive. New thoughts are blown like a spirit in the body of the organization and will save it from degeneration and destruction. In the present age, for survival and progress or even maintaining the present situation it is necessary to create and encourage creativity in organizations and also help to nurture the creative abilities of individuals in organizations for changing its structural body (1). For the creation and encouraging of creativity in organizations, several variables, such as structural or human, are effective (2).

However, structural variables are much more effective in the creation and nurturing of creativity, which could be generally categorized into two types of mechanical and organic structures. 

Mechanical structures are rigid and dependent on the type of machine on which the relations and functions are exactly specified and are not intended primarily for creativity and innovation (3). Organic structures are appropriate for the positions which are variable and uncertain. This kind of structure enjoys standards of living and non-hierarchical and robust behavior. Extensive communication is sufficient in it and it is appropriate to have freedom of creativity and innovation (4).

A structure that has already been agreed upon for the hospital management experts is the structure to provide the necessary stability within the hospital and community (5). The stability is the one which is capable of creating a suitable balance between hospital management and other members. The results of the research carried out by Omidi, Khabiri and Safari, entitled as “the relationship between organizational structure and creativity of director of physical education organization” suggests that the most important factors related to the decline of Creative Directors of Physical Education Organization, are focusing on individual decisions rather than participation in decision and measuring the degree of mismatch between job and education level (6, 7).

In a research conducted by Pierce and Delbacq (1997), it was suggested that organizational structure affects organizational innovation (8). They asserted that flexible structure would not only lead to the progress and development of the application of new ideas, but also these structures prove to be much more creative than the rigid ones (9).

Dale (1986) believed that bringing creative and innovative environment was perhaps the most important factor in ensuring the survival of the organization to provide new ideas.  Bundy (2002) in his study mentioned six major characteristics, including the freedom to express new ideas, a flat organizational structure, information management, and knowledge of the conflict, job requirements, qualifications and responsibilities for creative organizations. He believed that the above feature enables the organizations to optimize the creative process that will result in success. The only way organizations can be structured appropriately is to ensure the process of creativity (10, 11).

The main objective of this study was to determine the type of organizational structure (mechanical, organic) from the viewpoint of top and middle managers in 12 hospitals in Shiraz and also to carry out a comparative study of the amount of organizational creativity in each of these two types of structure.

## Methods

This is a cross-sectional and descriptive-analytic study. The setting in this study included 12 hospitals in Shiraz and the statistical population consisted of senior management (hospital administrator and director of nursing services) and intermediate managers (responsible for personnel, accounting and support services, managing laboratory and radiology) in teaching hospitals in Shiraz.

According to the statistical consultations and due to limited target population, the entire study population was considered as samples. Thus, the sample size in this study was 84. (12 hospitals and every hospital, n=7). The study of the organizational structure and organizational creativity is used as a measurement tool. 

The questionnaire was made by Robbins and Ivanovic (11) based on the Likert style and three dimensions were recognized, i.e. concentration, complexity and formality. Questions 1-5 were related to the recognition, 6-10 to the concentration, and 11-14 to the complexity. Also, the present scale aimed to determine the organizational structure of hospitals (organic, mechanical); mechanical hospitals obtain scores more than the average 46 and the organic hospitals obtain scores below average 46. In order to determine the validity of the organizational structure, comments of 4 university professors at Health Services Administration-medical (including one PhD and two Master of Health Services Administration-Health and master in health economics) were collected and after necessary editions, the validity was tested. To confirm the validity of the organizational structure questionnaire, first a preliminary study was carried out on six subjects and then Cronbach's alpha was used, yielding the result of 0.81.

The questionnaire used in this study is designed by Keramat Esmi in 2006 in order to evaluate the creativity of hospitals from the view point of managers; it contains 30 questions and has been designed in the Likrat style (7). The questionnaire measures six aspects including acceptance of change, tolerance for contradiction and external control measures. Questions 1-5 are about failure tolerance, 6-10 acceptance of ambiguity of the questions, 11-15 on encouragement of new ideas, questions, 16-20 about acceptance of change, 21-25 as to tolerating conflicts, and questions 26-30 are assigned to the low external control. To determine the validity of scale creativity, comments 4 Masters of Health Services Management - Medical (including one PhD and two Master of Health Services Administration-Medical and a master in health economics) were collected by the questionnaire. To confirm the reliability of the preliminary study, organizational creativity scale was performed on 20 samples. “Cronbach's alpha" coefficient was used for organizational creativity which proved to be 0.84. In this study, SPSS 14 (SPSS Inc, Chicago, IL, USA) was used to analyze the data, using t-test and Spearman's correlation coefficient.

## Results

According to the findings of present study, the highest score belonged to male managers who were the holder of B.S degree and the least to female managers holding M.A. and more. Data in Table 1 indicate that martyr Dastgheib hospital’s case managers have the perspective of the organic structure and those working in Zeinabieh and Ali Asghar Hospital believe in the mechanical structure.

**Table 1:** Information on the type of organizational structure from the perspective of hospital administrators

**Type of organizational structure** **Hospital**	**Organic structure**	**Mechanical structure**
	**N (%)**	**N(%)**
**Namazi**	2 (6)	5 (12)
**Faghihi**	4 (11)	3 (7)
**Zeinabieh**	5 (14)	8 (18)
**Dastgheib**	6 (17)	1 (4)
**Ali Asghar**	5 (14)	8 (18)
**Hafez**	4 (11)	3 (7)
**Chamran**	4 (11)	3 (7)
**Khalili**	2 (6)	4 (9)
**Ghotb e din**	2 (6)	4 (9)
**Ibn Sina**	1 (4)	5 (9)
**Total**	35 (100)	43 (100)

 The results shown in Table 2 and 3 indicate that among the six dimensions of organizational structure and creativity, there is a negative relationship between the levels of 0.01. This means the higher the organizational structure, the lower the organizational creativity.

**Table 2:** The relationship among organizational structure and organizational creativity, tolerance of failure, confusion and encourage the adoption of new ideas (creativity elements) from the perspective of directors

**Dependent** **variable** **Independent variable**	**Organizational creativity**			**Tolerance of failure**			**Confusion**			**Encourage the adoption of new ideas**		
	**N**	**r**	**p**	**N**	**r**	**p**	**N**	**r**	**p**	**N**	**r**	**p**
**Organizational structure**	78	-0.74	0.001	78	-0.62	0. 001	78	-0.65	0.0001	78	-0.45	0. 001

**Table 3:** The relationship among organizational structure and the acceptance of change, tolerance for contradiction and low external control elements (creativity) from the perspective of directors

**Dependent** **variable** **Independent variable**	**Acceptance of change**			**Tolerance for contradiction**			**Low external control elements**		
	**N**	**r**	**p**	**N**	**r**	**p**	**N**	**r**	**p**
**Organizational structure**	78	-0.36	0.001	78	-0.38	0.001	78	-0.51	0.001

 The findings suggest that there is a significant negative correlation between formal (organizational dimension) and overall organizational creativity. Our results indicate that the recognition of the acceptance of change, uncertainty and encourage adoption of the new theory, there is a significant negative relationship between external control low. Means that the force will increase creativity in general and the elements of creativity (accept change, embrace ambiguity, encourages new ideas and less control outside) decrease. Recognize the conflict between tolerances and tolerate failure after significant relationship was found.

Between the focus (organizational dimension) with overall organizational creativity and innovation in all aspects of this relationship indicates that there is a significant negative relationship is that increased focus, creativity and its dimensions are reduced.

Between the complexity (organizational dimension) with overall organizational creativity and innovation in six dimensions there is a significant negative correlation, which indicates that this relationship increasing complexity, creativity and its dimensions are reduced. Data in Table 4 indicate that the organic structure more creative creativity score in the mechanical structure.

**Table 4:** Innovation in organic structure and mechanics from the perspective of Directors

**Innovation** **Type of organizational structure**	**N**	**Min**	**Max**	**Mean±SD**
**Organic structure**	35	89	102	95.41±7.27
**Mechanical structure**	43	72	89	80.11±6.39
**Total**	78	80.5	95.5	94.26±6.83

## Discussion

The results of the study indicated a negative and significant relationship between organizational structure and creativity which designates the fact that with an increase in the rank of organizational structure, the amount of structural creativity decreases; this could be due to the fact that the organizational structure consists of three dimensions of formalization, centralization and complexity which can be reduced by increasing their creativity. Thus, according to the findings, the hospitals with a centralized structure are less creative than those with complex organization. These results are consistent with those of Nezam Shahidi (6), Pierce & Delbecq (8), and Senker (12). Correlation coefficient between organizational structure after failure tolerance, acceptance of ambiguity, encouraging new ideas, acceptance of change, tolerance for contradiction and low external control, all indicate that there is a significant negative relationship. Thus, the less centralized, formal and complex organizational structure of hospitals, i.e. those with organic organization, the higher the organizational creativity so that such hospitals accept failure, vagueness and uncertain ideas and encourage new ideas and the change. Conflicting ideas are presented in this hospital and their direct control and surveillance is low. In contrast, centralized structures, more complex and more formal ones, i.e. mechanical hospitals, are at a lower level n terms of the dimensions of organizational creativity.

It should be noted that martyr Dastgheib Hospital has the most organic structure and Zeinabieh and Ali Asghar Hospital has the mechanical structure. Moreover, there is a negative and significant relationship among the organizational creativity and its dimensions, which indicates the more concentrated structure organization and its six dimensions (bearing failure, accepting ambiguity, encouraging new ideas, acceptance of change, tolerance of contradiction and low external control) increase; this finding is consistent with those of Chandler (13) and Cushman (14).

There is a significant and negative relationship among the formal organizational creativity and four reception change, accepting ambiguity, encouraging new ideas and less of control outside. The obtained relationship indicates that the hospitals which strictly stick to official regulations, disregarding the personnel’s needs and interests, are increasing; in such hospitals, new ideas are welcome, external monitor is little, and vague ideas and change are accepted (11, 15). As a result, hospitals have been more creative and more flexible.

## Conclusion

Finally, it is recommended that managers should push their hospital toward the organic structure of the field for individual creativity and organization since a prerequisite for the survival of any organization and ability to compete is creativity and innovation in the current unstable situations. Limitations of this study are as follows:

1. Lack of adequate research in this area, especially when compared with medical universities and interpretation of results is difficult.
2. Failure to cooperate as well as lack of motivation to complete the questionnaire by some managers
3. Lack of timely and easy access to various hospital administrators.
4. Lack of quick and easy access to the text of the articles and external research done in this particular field of scientific sites.
